# Formation of a Novel Antagonistic Bacterial Combination to Enhance Biocontrol for Cucumber *Fusarium* Wilt

**DOI:** 10.3390/microorganisms13010133

**Published:** 2025-01-10

**Authors:** Fan Yang, Xin Wang, Huayan Jiang, Xiaoke Chang, Weiwei Chen, Gongyao Shi, Baoming Tian, Qiuju Yao

**Affiliations:** 1 Institute of Vegetable, Henan Academy of Agricultural Sciences, Graduate T&R Base of Zhengzhou University, Zhengzhou 450002, China; xiaoyuefuxiang@163.com (F.Y.); wx269152874@163.com (X.W.); jhyxb123@163.com (H.J.); cxk8802@163.com (X.C.); 2 School of Agricultural Sciences, Zhengzhou University, Zhengzhou 450001, China; weiwei_chen15134@zzu.edu.cn (W.C.); shigy@zzu.edu.cn (G.S.)

**Keywords:** synthetic microbial community, single-factor test, orthogonal test, response surface method, cucumber *Fusarium* wilt

## Abstract

*Paenibacillus polymyxa* strain PJH16, isolated and tested by our team, suppresses cucumber *Fusarium* wilt as an efficient biocontrol agent. For further investigation, the strain has been combined with two other *Bacillus strains* (*Bacillus velezensis* VJH504 and *Bacillus subtilis* JNF2) to enhance biocontrol ability, which formed high-efficiency microbial agents in the current study. The methodological target taken is based on achieving the optimal growth conditions of the combined microbial agents; hence, the medium composition and culture conditions were optimized through a single-factor test, orthogonal test and response surface methodology. Following this, the effectiveness of the microbial combination was assessed through pot experiments, which provided a theoretical foundation for the synthesis of microbial flora to significantly control cucumber Fusarium wilt. The results showed excellent compatibility, proving suitable for the proliferation and growth of *Paenibacillus polymyxa* PJH16, *Bacillus velezensis* VJH504, and *Bacillus subtilis* JNF2 strains together, specifically, when the inoculation amounts were adjusted to 4% of each. Using the single-factor test and orthogonal test analysis, the optimum composition of culture medium for the composite strain was identified as 3% glucose as the optimal carbon source, 2% yeast extract powder as the preferred nitrogen source, and 1% dipotassium hydrogen phosphate as the most suitable inorganic salt. Furthermore, the optical density (OD_600_) of the composite strain solution reached its highest level at 3.16 under the following culture conditions: inoculation volume of 200 µL, 171 rpm culture speed, 21.6 h culture time, 30 °C cultural temperature, and an initial pH of 7.0. The pot experiment demonstrated that the mixed bacterial solution achieved a relative control efficacy of 93.4% against cucumber *Fusarium* wilt, which was significantly superior to that of single- strain or pesticide treatment, and also promoted cucumber growth. In summary, the microbial flora synthesized by the three *Bacillus* strains displayed a high bacterial concentration, following the optimization of culture conditions, and exerted remarkable control and growth-promoting effects on cucumber *Fusarium* wilt. This finding holds great significance for future developments of composite microbial agents.

## 1. Introduction

The cucumber is one of the top ten vegetables in the world, which is widely planted all over the world [1]. The yield of cucumber may be seriously reduced due to infection with *Fusarium oxysporum* f. sp. *Cucumerinum*. This pathogen can cause cucumber *Fusarium* wilt, and its symptoms include necrosis at the lesion, vascular bundle and root wilt, eventually leading to plant death [2]. Therefore, cucumber *Fusarium* wilt, also known as ’plant cancer’, generally leads to a 10–30% reduction in cucumber yield, and even 80–90% in severe cases [3]. It is a devastating global soil-borne disease. The disease can occur in all stages of cucumber growth. Once the plant is infected, the pathogen will be difficult to remove, and its survival period can last up to 20 years [4]. Over the past few decades, the control of soil-borne diseases has generally relied on the breeding of resistant varieties and the use of chemical fungicides. However, in the absence of dominant genes, the development of resistance quality may be significantly hindered [5]. The use of chemical fungicides will cause certain harm to the environment and humans [6]. It has been reported that the use of chemical reagents can lead to changes in microbial community structure, which may reduce the activity of beneficial microorganisms and promote the growth of drug-resistant pathogens [7]. Therefore, microbial therapy has emerged as a simple and effective alternative to disease control by cutting off the connection between plant hosts and pathogens. Most microorganisms are capable of producing and releasing one or more antimicrobial substances, which can inhibit or kill bacteria and other pathogens in a certain amount [8]. Bacteria with the biocontrol ability can produce bacteriocins, antibiotics and other substances to inhibit the growth of pathogens [9]. Bacteria such as *Bacillus* [10], *Pseudomonas* [11] and *Streptomyces* [12] have been demonstrated to display significant efficacy in controlling plant diseases. Meyer et al. found that *Pseudomonas* F9-7 and *Bacillus* F9-2 and F9-12 isolated from forestry compost could inhibit the mycelial growth of *Sclerotinia sclerotiorum* and effectively control white mold of carrots [13]. Abdelhalim et al. have proven that *Bacillus subtilis* and *Streptomyces terminalis* have significant inhibitory effects on *Botrytis cinerea*, both in vitro and in vivo. In the future, they have the potential to serve as biological agents for replacing chemical fungicides in the management of apple gray mold [14]. Currently, the majority of research in the field of biological control focuses on utilizing single biocontrol microorganisms to manage plant diseases. However, this approach faces several challenges, including the reliance on a single biocontrol mechanism, high environmental dependence, limited persistence, and susceptibility to external conditions. de Boer et al. demonstrated that the disease suppression effect of the *Pseudomonas putida* strains WCS358 and RE8, when mixed with soil, was significantly better than that of single-strain treatments. Moreover, when either *Pseudomonas putida* strains WCS358 or RE8 failed to control plant diseases individually, the combination of the two strains still effectively managed the diseases [15]. *Bacillus* sp. Og2 and *Pseudomonas fluorescens* Oq5, either individually or in combination, were effective in controlling olive knot disease. However, plants treated with the combined bacterial solution showed a significantly greater reduction in disease severity (89.58%) [16]. The combination of multiple microbial agents can complement each other through the unique characteristics of biocontrol bacteria, thereby enhancing the stability and effectiveness of their control outcomes.

During the use of single biocontrol bacteria, they may be eliminated by the competition of pre-existing resident microbial flora resources or direct interference competition, resulting in single beneficial bacteria often being unable to achieve stable disease suppression in the field due to their weak rhizosphere colonization ability. In the complex ’arms race’ between plants and pathogens, the rhizosphere soil microbiome is the first line of defense against pathogenic invasion of plant roots. The inoculation of beneficial microbiome can first form a biological barrier around the crop rhizosphere, and then cut off the connection between the plant host and the pathogen, thus resisting the occurrence of soil-borne diseases and insect pests. Secondly, the dynamic balance and stability of soil micro-ecological systems are maintained by recruiting functional microorganisms. Again, they induce crops to produce immune function to improve disease resistance, and then form a lasting inhibition of disease soil. Sundaramoorthy et al. found that compared with single biocontrol strains, the combination of *Bacillus subtilis* EPCO16,EPC5 and *Pseudomonas fluorescens* Pf1 bacterial strains can produce a variety of antibiotics, continuously inhibit the growth of *Fusarium solani* mycelium, and can control pepper *Fusarium* wilt by inducing ISR [17]. In recent years, it has become a new technology and method to reduce the application of chemical pesticides and improve soil health by adding microbial groups to facility soil, cultivating specific functional microbial groups, and constructing disease-suppressing rhizosphere soil micro-ecological environments. For example, the control effect on the root-knot nematode of lettuce was 34.9% when using *Purpureocillium lilacinus* and *Ochrobactrum pseudogriseon* as compound microbial agents [18], and *Streptomyces* Ahn75 and *Bacillus amyloliquefaciens* CWJ2 were isolated from healthy rice by Hu et al. The compound microbial agent composed of strains Ahn75 and CWJ2 was used to treat rice panicle blast, and the control effect was 52.16% [19]. Wang et al. verified, through soil pot experiments, that the control effect of compound microbial agents (*Streptomyces* WL-4 and *Bacillus* CW-02) on tomato bacterial wilt was 45.58%, which was better than that of the single application of microbial agents WL-4 and CY-1 and could improve the bacterial community structure in the tomato rhizosphere [20]. Studies have also shown that the compound fermentation of *Actinomycetes* FX81 and FX28 has a pot control effect of 82.03% on tomato bacterial wilt, which is 8.13% and 18.6% higher than that of a single strain [21]. Therefore, the advantages of compound microbial inoculants, compared with single microbial inoculants, are their stronger persistence and more functions, which can improve the control effect of plant diseases [22].

The *Bacillus* genus encompasses a vast diversity of species, which are widely distributed and can inhibit a variety of pathogens. It is an ideal biocontrol microorganism [23]. The common species of *Bacillus* include *Bacillus subtilis* [24], *Bacillus amyloliquefaciens* [25], *Bacillus velezensis* [26] and *Paenibacillus polymyxa* [27]. *Bacillus* can produce heat-resistant and stress-resistant spores, which is superior to other biocontrol agents in terms of stability and compatibility with chemical pesticides [28], and can effectively control cucumber *Fusarium* wilt [29], wheat seedling blight disease [30], rice blast [31], potato scab disease [32], wheat root rot [33] and other plant diseases. *Bacillus* has significant prevention and control effects on pathogens of various plant diseases; they can produce many secondary metabolites to dissolve the cell wall or cell membrane of pathogens, thereby interfering with their division, affecting energy metabolism and protein synthesis. In order to achieve the effect of controlling the growth and reproduction of pathogens [34]. *Bacillus* can dissolve a variety of pathogens by secreting chitinase [35], protease, cellulose degrading enzymes [36] and so on [37]. Guleria et al. found that *Bacillus* sp. SP1 can inhibit the growth of *Fusarium oxysporum* by secreting proteases [38]. Zhan et al. isolated seven strains of bacteria with antagonistic effects on *Fusarium* wilt from tomato soil. Among them, B-5 and LYT-5 were identified as similar species of *Bacillus amyloliquefaciens* and *Bacillus velezensis* by a phylogenetic analysis of 16S rRNA. Both strains can inhibit the growth of *Fusarium oxysporum* and have significant antagonistic effects on tomato *Fusarium* wilt [39]. Due to its significant biocontrol effect, many studies have evaluated the biocontrol effect by compounding several *Bacillus* strains to obtain efficient and stable biocontrol agents. Chen et al. showed that the inhibition rate could reach 59.46% when *Bacillus megaterium* Y-30 and *Bacillus amyloliquefaciens* CM3 were combined in a ratio of 2:1, which was better than that of a single strain [40].

In this study, three biocontrol strains, *Bacillus velezensis* VJH504 [41], *Paenibacillus polymyxa* PJH16 [42] and *Bacillus subtilis* JNF2 [43], which were screened in the rhizosphere soil of cucumber with *Fusarium* wilt in the early stage and had a strong control effect on cucumber *Fusarium* wilt, were used for compatibility determination, strain ratio, inoculation ratio, inoculation amount, and culture condition screening to synthesize the microbial flora with the best growth status. Finally, its control effect was evaluated. The aim is to provide new ideas and technical support for the later development of new composite microbial agents to target and accurately control cucumber *Fusarium* wilt and regulate microbial community conditions to solve potential obstacles in future production.

## 2. Materials and Methods

### 2.1. Preparation of Seed Liquid and Tested Strains

Biocontrol Strains: They was isolated and preserved by the Vegetable Research Institute of Henan Academy of Agricultural Sciences, and each strain was reported. The rhizosphere soil of healthy cucumbers from a high-incidence area of cucumber *Fusarium* wilt in Beiwang Village, Luolong District, Luoyang City, Henan Province, China, was randomly sampled. Through isolation, purification and antagonistic screening from soil, three strains with significant biocontrol effects were obtained, which were *Bacillus velezensis* VJH504, *Paenibacillus polymyxa* PJH16 and *Bacillus subtilis* JNF2. The above strains were cryopreserved at −80 °C in an LB medium containing 20% glycerol.

Fungal pathogen: *Fusarium oxysporum* f. sp. *Cucumerinum* (*FOC*) 72-1 were isolated and preserved by the Vegetable Research Institute of Henan Academy of Agricultural Sciences. The strain was routinely cultured on a potato dextrose agar (PDA) medium at 28 °C.

The strains of *B. velezensis* VJH504, *P. polymyxa* PJH16 and *B. subtilis* JNF2 were streaked on LB agar plates and incubated at 30 °C for activation. Subsequently, the activated strains were selected using a sterile inoculation loop and transferred to 250 mL Erlenmeyer flasks containing 100 mL of LB borth. The cultures were incubated at 30 °C with shaking at 180 rpm for 36 h. The OD_600_ value of the resulting seed culture was adjusted to 0.8.

### 2.2. Determination of Strain Compatibility

The three strains were cross streaked on an LB agar and incubated at 30 °C for 24 h to observe the presence of any bacteriostatic band between the antagonistic strains. The absence of a bacteriostatic band indicates good compatibility between the strains, which allows for them to be further studied for their potential use in the microbial management of cucumber *Fusarium* wilt.

### 2.3. Initial Inoculation Concentration Ratio of the Strains

An L_9_ (3^4^) orthogonal experiment with three factors and three levels was conducted to determine the optimal inoculation concentration of each strain. The factors included the inoculation concentrations of the three strains, while the measurement indices were the OD_600_ value of the mixed bacterial culture and the inhibition rate against FOC. The inoculation levels were optimized using seed cultures at 4%, 8%, and 12% (*v*/*v*). The seed cultures of the three strains listed in Table 1 were mixed in a 250 mL Erlenmeyer flask containing 100 mL of LB broth, based on the proportions outlined in the orthogonal experiment design. After 36 h of shaking incubation at 30 °C and 180 rpm, the OD_600_ value was measured, and the antagonistic assay against FOC was performed. The inhibition rate (%) was calculated as (colony diameter of control pathogen—colony diameter of treated pathogen)/colony diameter of control pathogen × 100%.

### 2.4. Screening of Carbon Sources, Nitrogen Sources, and Inorganic Salts in the Basal Medium

Single-factor experiments were conducted for the carbon sources, nitrogen sources and inorganic salts to determine the optimal medium composition. Based on an LB medium, the carbon sources were individually optimized using glucose, maltose, fructose, sucrose and soluble starch. The nitrogen sources were optimized using beef extract, peptone, yeast extract, tryptone, soybean peptone and ammonium sulfate, respectively. The inorganic salts were optimized using sodium chloride, disodium hydrogen phosphate, dipotassium hydrogen phosphate, calcium carbonate, magnesium sulfate and zinc sulfate, respectively. Each carbon source, nitrogen source, or inorganic salt constituted a separate treatment, with the LB medium serving as the control. Each treatment was replicated three times, and the OD_600_ value of the mixed bacteria culture, along with the inhibition rate against *FOC*, were measured.

### 2.5. Optimization of Fermentation Basic Medium for Synthetic Microbial Communities

The carbon sources, nitrogen sources, and inorganic salts were screened at five different concentrations (0.3%, 0.5%, 1.0%, 2.0%, 3.0% *w/v*) using a three-factor, five-level L_25_ (5^6^) orthogonal experiment to determine the optimal ratio, as shown in Table 2.

### 2.6. Optimization of Culture Conditions for Synthetic Microbial Communities

The optimal liquid medium was used to optimize single-factor conditions, including inoculum size, cultural temperature, agitation speed, fermentation time, and initial pH for the mixed strains. The inoculum sizes tested were 50, 100, 150, 200 and 250 µL. The fermentation times were 12, 15, 18, 21 and 24 h. The culture temperatures were 20, 25, 30, 35 and 38 °C, and the agitation speeds were 120, 150, 180, 200 rpm. The initial pH levels were 5.0, 6.0, 7.0, 8.0 and 9.0. Each treatment was replicated three times, and the OD_600_ value of the fermentation broth, along with the inhibition rate against *FOC*, were measured.

Based on these results, the three most significant influencing factors were selected, with the OD_600_ value of the fermentation broth as the response variable. A response surface methodology (*n* = 3) was then used to further optimize the culture conditions of the mixed strains, determining the final optimal culture parameters.

### 2.7. Pot Control Effect Determination

Cucumber seeds of Bojie 616 were soaked in a spore suspension containing 10^7^ cfu·mL^−1^ FOC strain 72-1 for 30 min, germinated, and then sown in 40-hole trays. After 5 days, the roots were treated with biocontrol agents or chemical pesticides. The treatments included 0.1% Hymexazol, *B. velezensis* VJH504 (OD_600_ = 0.8), *P. polymyxa* PJH16 (OD_600_ = 0.8), *B. subtilis* JNF2 (OD_600_ = 0.8), and a mixed bacterial solution (OD_600_ = 0.8), with clear water irrigation serving as the control. Each treatment received 10 mL of solution per hole for root irrigation, with three replicates per treatment and 200 plants per replicate. Greenhouse conditions were maintained, and the data were collected for statistical analysis 20 days after inoculation.

The disease severity was rated as follows: 0 = normal growth with no symptoms; 1 = yellowing of cotyledons without wilting; 2 = wilting of cotyledons; 3 = wilting of cotyledons and true leaves or stunting of the plant; 4 = complete plant death. The disease index and control efficacy were calculated using the following formulas:

Disease index = [Σ (Total number of plants of each disease grade × The representative value of each level)/(Investigate the total number of plants × Highest-level value)] × 100% [44].

Control effect (%) = [(Control disease index—treatment disease index)/Control disease index] × 100% [45].

### 2.8. Data Statistics and Analysis

The experimental data were analyzed using a one-way analysis of variance (ANOVA) in SPSS software (v21.0). Duncan’s multiple range test was used for mean comparisons, with the significance set at *p* ≤ 0.05.

## 3. Results

### 3.1. Compatibility Analysis

**Figure 1:**
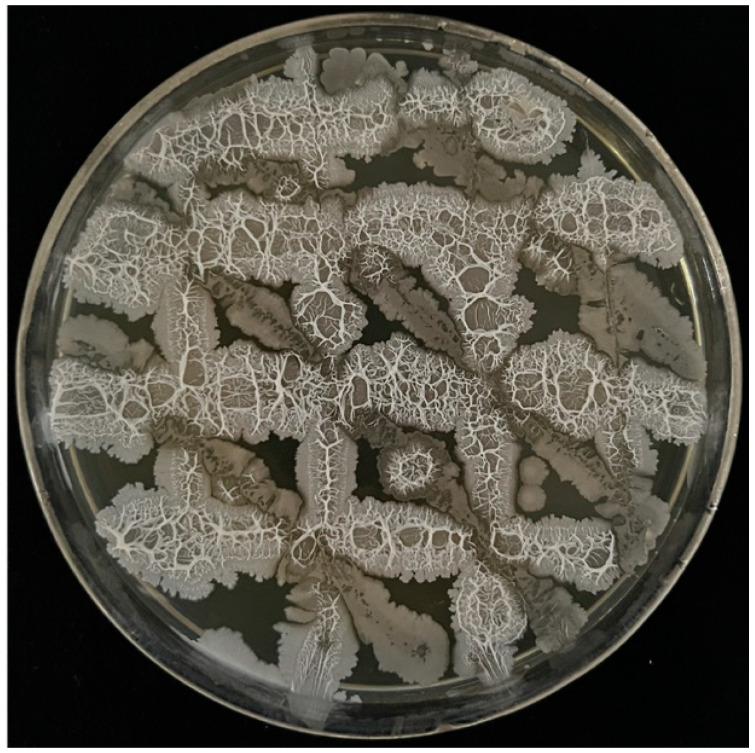
Compatibility of *B. velezensis* VJH504, *P. polymyxa* PJH16 and *B. subtilis* JNF2.

As shown in Figure 1, the strains *B. velezensis* VJH504, *P. polymyxa* PJH16 and *B. subtilis* JNF2 exhibited normal growth in the LB medium. No antagonistic or inhibition zones were observed between the strains, indicating that they are mutually compatible. This compatibility suggests that these strains can be combined to form a composite microbial agent for further research, which also means that *Bacillus velezensis*, *Paenibacillus polymyxa* and *Bacillus subtilis* can act synergistically on *Fusarium oxysporum*, and it is expected to obtain a good biocontrol effect.

### 3.2. Analysis of Initial Concentration Ratio of Strain Inoculation

Table 3 shows the results of a single-factor and orthogonal analysis of the inoculation concentrations. When the inoculation rates for *B. velezensis* VJH504, *P. polymyxa* PJH16, and *B. subtilis* JNF2 were all set to 4%, the cell density (OD_600_) of the mixed culture reached 1.457, the highest recorded value. Furthermore, this mixture achieved a 93.78% inhibition rate against *FOC* 72-1, making this concentration ratio the most effective for promoting the growth of mixed strains and biocontrol efficacy.

### 3.3. Optimization Results of Fermentation Basic Medium for Synthetic Microbial Communities

#### 3.3.1. Effects of Carbon, Nitrogen Sources and Inorganic Salts on Growth and Antibacterial Activity of Mixed Strains

**Figure 2:**
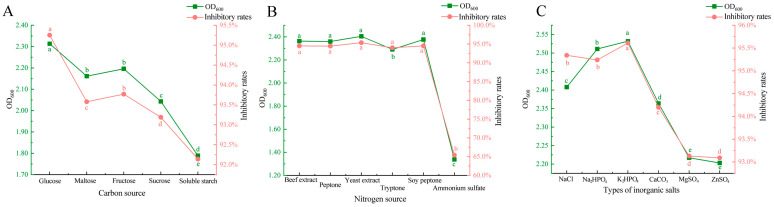
Effects of different carbon sources, nitrogen sources and inorganic salts on bacterial density and antibacterial activity of the compound microbiome. (**A**) The effects of different carbon sources on the cell density and antibacterial activity of the compound microbiome. (**B**) The effects of different nitrogen sources on the cell density and antibacterial activity of the compound microbiome. (**C**) The effects of different inorganic salts on the cell density and antibacterial activity of the compound microbiome. Different letters (a–e) in the figure indicate significant differences (*p* < 0.05).

Carbon source, nitrogen source and inorganic salt play vital roles in the growth of microorganisms. They are not only an important part of microbial cells, but also participate in and affect cell synthesis, energy conversion, metabolic pathways and so on. The results indicated that glucose, as a carbon source, resulted in the highest OD_600_ value of the mixed strain fermentation broth and achieved the most effective inhibition of *FOC* 72-1 mycelium. Conversely, when soluble starch was used as the carbon source, the OD_600_ value of the fermentation broth was the lowest, and the inhibition rate of *FOC* 72-1 mycelium was the least effective (Figure 2A).

Regarding the nitrogen sources, yeast extract significantly enhanced both the growth of the mixed strain fermentation broth and the inhibition rate of *FOC* 72-1. In contrast, the use of ammonium sulfate as the nitrogen source resulted in the lowest cell growth and the poorest inhibition of *FOC* 72-1 (Figure 2B).

For inorganic salts, dipotassium hydrogen phosphate (K_2_HPO_4_) proved to be the most effective. It supported the highest growth and reproduction rates of the three strains, as indicated by the highest OD_600_ value. Furthermore, the inhibitory effect on the mycelium of *FOC* 72-1 was markedly superior to that observed with other inorganic salts (Figure 2C).

#### 3.3.2. Effects of Different Ratios of Carbon and Nitrogen Sources and Inorganic Salts on the Growth and Antibacterial Activity of Mixed Strains

An orthogonal combination test was conducted to evaluate the effects of varying the ratios of glucose, yeast extract powder, and dipotassium hydrogen phosphate as a carbon source, nitrogen source, and inorganic salt, respectively, in an LB liquid medium. This test included three factors at five levels.

The results demonstrated significant variations in the growth and antibacterial activity of the mixed strains, depending on the proportions of these components. Specifically, when glucose, yeast extract powder, and dipotassium hydrogen phosphate were present at concentrations of 3%, 2%, and 1%, respectively, optimal growth was achieved. Under these conditions, after 36 h of incubation at 30 °C and 180 rpm, the mixed strains exhibited the highest OD_600_ value and the greatest inhibition rate against *FOC* 72-1 (Table 4).

### 3.4. Optimization Results of Culture Conditions of Synthetic Microbial Communities

#### 3.4.1. The Effect of Single Factor Optimization on the Growth and Antibacterial Activity of Mixed Strains

1. Inoculation amount

**Figure 3:**
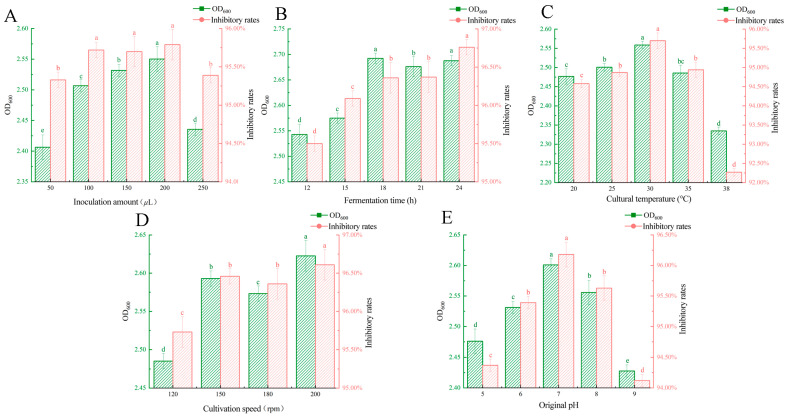
The influence of different single factors on the bacterial density and antibacterial activity of the compound microbiome. (**A**) The effect of inoculation amount on the cell density and antibacterial activity of the compound microbiome. (**B**) The effect of fermentation time on the cell density and antibacterial activity of the compound microbiome. (**C**) The effect of cultural temperature on the cell density and antimicrobial activity of the compound microbiome. (**D**) The effect of cultivation speed on the cell density and antibacterial activity of the compound microbiome. (**E**) The effect of original pH on the cell density and antimicrobial activity of the compound microbiome. Different letters (a–e) in the figure indicate significant differences (*p* < 0.05).

The inoculation volume significantly influences the growth and proliferation of mixed strains. The experimental results are shown in Figure 3A. The OD_600_ value of the mixed bacteria initially increased with higher inoculation volumes but subsequently declined. At an inoculation volume of 200 µL, both the OD_600_ value and the inhibition rate of *FOC* 72-1 were maximized after fermentation for 36 h. This was significantly higher than those of the initial inoculation amount of 50 µL and 250 µL, but there was no significant difference with the initial inoculation amount of 100 µL and 150 µL. Based on these findings, the optimal initial inoculation volume for mixed strains was determined to be 100–200 µL.

2. Fermentation time

The duration of culture significantly influences microbial growth rates. In this study, the OD_600_ value of the mixed bacterial culture initially increased with prolonged incubation time but then began to decrease (Figure 3B). At 18 h and 24 h, the OD_600_ values were significantly higher than that at other time points, with a peak value of 2.591. Moreover, culture duration notably affected the inhibition rate of *FOC*. The bacterial mixture exhibited the highest inhibitory effect on *FOC* at 18 and 24 h of shaking incubation, achieving a maximum inhibition rate of 96.79%. Therefore, the optimal incubation time for the mixed bacterial culture was preliminarily determined to be 18–24 h.

3. Cultural temperature

The level of culture temperature affects important physiological and biochemical processes, such as enzyme activity. The results showed that when the culture temperature was 30 °C, the OD_600_ value of the mixed bacteria solution and the inhibition rate of *FOC* reached the maximum and were significantly higher than those observed under other temperature treatments (Figure 3C).

4. Cultivation speed

The growth of bacteria is affected by the culture speed, which is mainly achieved by adjusting the content of dissolved oxygen in the medium. In this study, the OD_600_ value of the mixed strain increased first and then decreased with the increase in the culture speed. When the culture speed was 150–200 rpm, the OD_600_ value and the bacteriostatic rate had a maximum value of 2.622 (Figure 3D). Therefore, it is preliminarily determined that the culture speed range of the mixed flora oscillation culture is 150–200 rpm.

5. Original pH

As a key environmental factor, pH value directly affects the core physiological functions of bacteria, including the regulation of enzyme system activity and cell membrane permeability, thus having a profound impact on the normal growth cycle of bacteria. In this study, it was found that pH had a significant effect on the OD_600_ value of the mixed bacterial solution and the bacteriostatic rate of *FOC* 72-1. The OD_600_ value of the bacterial solution and the inhibition rate of *FOC* hyphae showed a trend of increasing and then decreasing. When the pH was 7.0, the cell density value and the inhibition rate of the pathogen hyphae were the highest, which was significantly different from other treatments (Figure 3E). Therefore, pH 7.0 is the best pH for mixed strain culture.

#### 3.4.2. Experimental Analysis of Response Surface Optimization of Culture Conditions

The single-factor experiment identified the optimal conditions for the fermentation of the mixed bacterial strains: a temperature of 30 °C, an initial pH of 7.0, an inoculum size of 100–200 µL, a culture speed of 150–200 rpm, and a culture time of 18–24 h. A statistical analysis using SPSS software (V 17.0) showed that inoculum size, culture speed, and culture time were significant factors influencing fermentation, with *p*-values < 0.05. Consequently, these parameters were selected for further optimization using a Box–Behnken design in Design Expert software (V 8.0.6).

The OD_600_ value of the mixed bacterial solution was used as the response value, and the response surface experimental design of three factors and three levels was carried out to determine the optimal fermentation conditions (Table 5).

The inoculation amount (A), culture speed (B) and culture time (C) were used as independent variables, and the OD_600_ value was the response value. The results of Table 6 were fitted using a multiple regression equation through Design Expert (V 8.0.6) software. The prediction model of the multivariate quadratic regression equation was obtained: OD_600_ = −114.94287 + 0.040150A + 0.52191B + 6.53470C + 2.31400E − 004AB − 3.63333E − 004AC − 5.94000E − 003BC − 2.13010E − 004A^2^ − 1.28004E − 003B^2^ − 0.12598C^2^.

The obtained regression equation prediction model was subjected to variance analysis and significance testing. As shown in Table 7, the model was extremely significant (*p* = 0.0023 < 0.01). The lack of fit term had a *p* of 0.107 (*p* > 0.05), indicating that the model fits the experimental data well. The interactions between factors varied, with the significance order being AC (*p* = 0.7935) > AB (*p* = 0.1922) > BC (*p* = 0.0616). The coefficient of determination (R^2^) of the model was 0.9343, indicating that the model explains 93.43% of the variability. The adjusted R^2^ value of 0.8498 suggests that the model has a strong fit with minimal experimental error, effectively reflecting the changes in the response variable.

The results indicate that the model shows good correlation and can reliably predict the response values under different conditions, thereby determining the optimal fermentation parameters. Additionally, the first-order terms, A, B and C, of the model had significant effects (*p* < 0.05), and the second-order terms, B2 and C2, had extremely significant effects (*p* < 0.01). According to the F value and *p* value of each factor in Table 7, it can be concluded that the order of their influence on OD_600_ density value is as follows: inoculation amount (A) > culture time (C) > culture speed (B).

**Figure 4:**
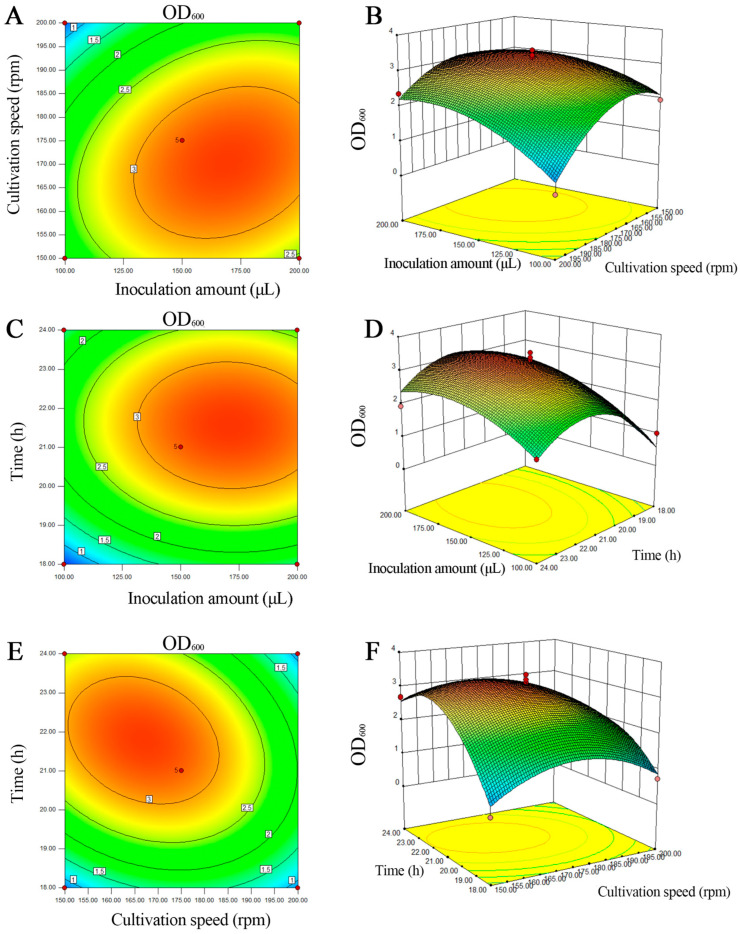
Response surface and contour map of the interaction of different culture factors on OD_600_ value. (**A**) Contour map of the effect of the interaction between inoculation amount and culture speed on OD_600_ value. (**B**) Response surface of the effect of the interaction between inoculation amount and culture speed on OD_600_ value. (**C**) Contour map of the effect of inoculation amount and culture time on OD_600_ value. (**D**) Response surface of the effect of inoculation amount and culture time on OD_600_ value. (**E**) Contour map of the effect of culture speed and time on OD_600_ value. (**F**) Response surface of the effect of culture speed and time on OD_600_ value.

According to the data in Table 7, quadratic multiple regression fitting was carried out, and the contour map and 3D surface maps generated from the regression equation are shown in Figure 4. The results showed that the contour lines for inoculum size and culture time, inoculum size and culture speed, culture time and culture speed were elliptical, while the corresponding 3D surface maps were steep, indicating significant interactions between these factors.

The response surface curve of the factor interactions revealed the presence of extreme points. Through an analysis using Design Expert (V 8.0.6) software, the optimal culture conditions were obtained as follows: inoculum size of 200 µL, rotation speed of 171 rpm, culture time of 21.6 h, culture temperature of 30 °C, and an initial pH of 7.0. Under these optimized conditions, the OD_600_ density of the mixed bacterial solution reached a maximum value of 3.16.

#### 3.4.3. Determination of the Optimal Fermentation Conditions of Mixed Strains and Model Validation

Considering practical operation conditions, the optimal fermentation parameter adjustment results are shown in Table 8: an initial inoculum size of 200 µL, culture speed of 171 rpm, culture time of 22 h, cultural temperature of 30 °C, and an initial pH of 7.0. Under these conditions, three parallel verification experiments were carried out, yielding an OD_600_ value of 3.17 for the mixed bacterial culture. The small discrepancy between the actual and predicted values indicates a high degree of model fit, confirming the accuracy and reliability of the model.

### 3.5. Evaluation of the Effect of Mixed Bacterial Solution on Cucumber Fusarium Wilt

**Figure 5:**
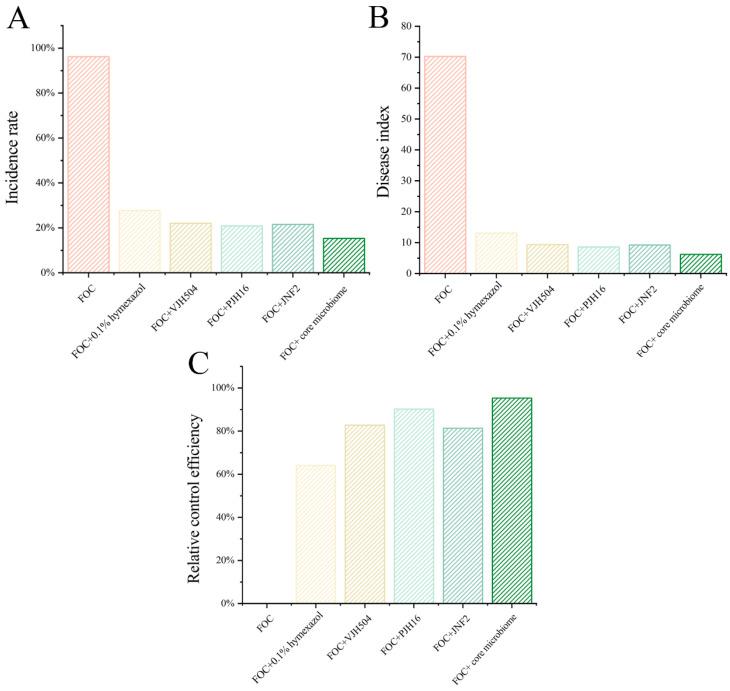
Potted control effect of the compound microbiome. (**A**) The incidence of cucumber seedlings after using single or compound bacterial solution. (**B**) The disease index of cucumber seedlings after using single or compound bacterial solution. (**C**) The relative control effect of single or compound bacterial solution on cucumber seedlings.

**Figure 6:**
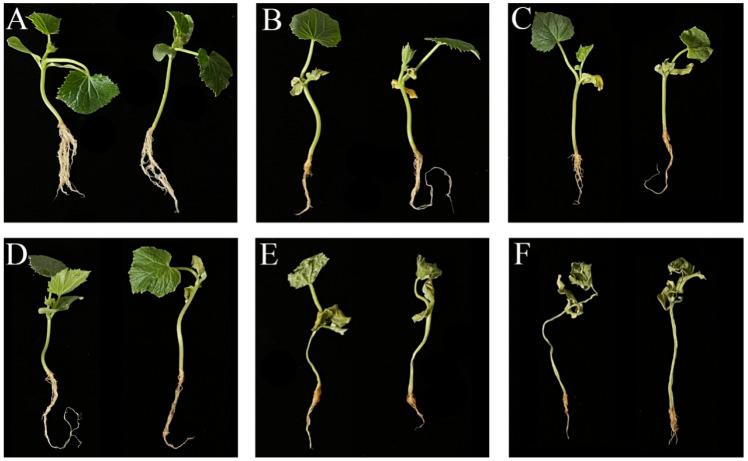
Control effect of the compound microbiome. (**A**) The control effect of the compound microbiome. (**B**) The control effect of *B. velezensis* VJH504. (**C**) The control effect of *P. polymyxa* PJH16. (**D**) The control effect of *B. subtilis* JNF2. (**E**) The control effect of hymexazol. (**F**) Cucumber seedlings inoculated with *FOC* only.

To evaluate the efficacy of the synthetic microbial flora in the prevention and control of cucumber *Fusarium* wilt, the control effect of mixed bacterial solution on cucumber *Fusarium* wilt was assessed using plug seedling inoculation with *FOC* and biocontrol bacteria. The results, as shown in Figure 5 and Figure 6, demonstrated a significant protective effect, with a relative control efficacy of 93.4%, which was significantly higher than that of individual strain or chemical pesticide treatments. Furthermore, the mixed bacterial solution promoted cucumber growth. These findings further validate the reliability of the response surface analysis method.

## 4. Discussion

The rhizosphere is a special micro-ecological system, which contains soil, plants, microorganisms, etc. It is the first threshold for soil-borne pathogens to enter crops [46]. There is a close correlation between rhizosphere soil microbiome (group) and soil-borne pathogens [47]. It is the first line of defense against the pathogenic invasion of plant roots. Biological control is a method that can effectively control pathogens and pests to increase crop yield [48]. In biological control, in addition to the classic method of using a single inoculum, the use of synthetic microbial communities is a promising method to promote plant growth, enhance disease resistance and improve stress resistance. A large number of studies have shown that the combination of a variety of biocontrol bacteria can effectively control *Fusarium* wilt [49]. In the design of synthetic microbiome, people try to design plant-specific synthetic microbiome through the excavation of rhizosphere core microbial groups and the regulation of core microbial interactions, so as to reshape the plant rhizosphere microbial community and enhance the growth metabolism and stress resistance of plants. Santhanam et al. constituted five local strains into a mixed flora and found that the mixed bacteria had a significant inhibitory effect on tobacco *Fusarium* wilt [50]. Cheng et al. reported that the compound strain composed of *Bacillus velezensis* WZ-37, *Bacillus subtilis* WXCDD105 and *Bacillus amyloliquefaciens* SS could not only reduce the water loss rate of tomato fruit, but also had a good inhibitory effect on the occurrence of tomato gray mold [51]. However, multi-strain microbiome assembly is a challenging and complex study, which usually requires the combination of two or more strains, and requires a consideration of the number of strains, the ability of each strain to inhibit pathogens, and the ecological interactions between strains [52]. Therefore, the selection of strain sources and the optimization of culture conditions are very important conditions for the construction of a synthetic microbiome. Li et al. isolated and screened cellulose-degrading bacteria from a mixture of rotten wheat straw residues to construct a microbial system ADS-3. At 3 d and 11 d, the activities of xylanase and cellulase could reach 1.32 and 0.15 U·mL^−1^ and the degradation of the straws was obvious [53]. Yan et al. determined the optimal culture conditions by a single factor test and orthogonal test. The relative control effect of the optimized antagonistic combination *Streptomyces rimosus* GQ-17, *Streptomyces diastaticus* YH-91 and *Streptomyces pactum* YH-23 was 65.89%, which was higher than that of the single strain (19.94%, 25.20%, 42.88%) and Shenqinmycin (39.50%) [54].

Existing studies have shown that secondary metabolites secreted by biocontrol bacteria can effectively control the growth of pathogen hyphae, spore formation and germination [55], and it can regulate the salicylic acid signal by producing organic compounds, thereby inducing plant systemic resistance to resist diseases [56]. It can also directly inhibit the growth of pathogens by producing a variety of hydrolases and antimicrobial lipopeptides that act on the cell wall of pathogens [57]. Although single biocontrol bacteria can effectively control plant diseases, single strain and single function biocontrol bacteria are gradually becoming unable to meet the needs of agricultural development [58]. The combination of multiple biocontrol strains may provide a variety of disease resistance mechanisms and stable community structure due to the distant genetic relationship between different strains and the greater biological specificity, thus playing a more stable and efficient role in controlling plant diseases. In this study, three strains with significant biocontrol ability were screened from the rhizosphere soil of healthy cucumber in the high incidence area of cucumber *Fusarium* wilt: *Bacillus velezensis* VJH504, *Paenibacillus polymyxa* PJH16 and *Bacillus subtilis* JNF2. According to the results of previous whole genome sequencing, the above strains contain Surfactin, Paenibacillin and Glycocin synthesis gene clusters, compared with other biocontrol bacteria, and these gene clusters have the effect of inhibiting the growth and development of fungi. At the same time, the differences in these synthetic gene clusters may also lead to differences in their ability to control plant diseases. Moreover, the above three strains can also produce indole acetic acid (IAA) and siderophores and have enzyme activities, such as those performed by β-glucanase, protease and amylase. While inhibiting the growth of pathogens, it can also promote the growth of cucumber seedlings. In this study, *Bacillus velezensis* VJH504, *Paenibacillus polymyxa* PJH16 and *Bacillus subtilis* JNF2 not only had good compatibility, but also found that the biocontrol effect of cucumber seedlings treated with compound bacterial solution was significantly better than that of cucumber seedlings treated with a single strain or pesticide, and the compound strain also had a promoting effect on cucumber seedlings. *Bacillus* is often an important biocontrol microbial resource because of its stable physical and chemical properties, strong reproductive ability, abundant population in soil and wide antibacterial spectrum [59]. The strains selected in this study were all *Bacillus*, and the compatibility between the same genus strains may be stronger. Existing studies have shown that Surfactin can be used as an interspecies recruitment factor [60]. Luzzatto-Knaan et al. showed that *Bacillus subtilis* secreted Surfactin to recruit *Paenibacillus dendritic* to its niche [61]. The above results are consistent with the conclusion that *Bacillus velezensis* VJH504, *Paenibacillus polymyxa* PJH16 and *Bacillus subtilis* JNF2 have good compatibility in this study. Therefore, an appropriate amount of bacteria composed of different strains can enhance the inhibitory effect on the growth of pathogens. This may be due to the fact that the secondary metabolites produced by different antagonistic strains can synergistically inhibit pathogens. Due to the different molecular mechanisms and modes of action of compounds produced by different strains, the bacterial community composed of different strains could produce more kinds of chemicals, thereby increasing the total antibacterial activity of the combined flora. Next, we will study the synergistic mechanism of the strains.

The synthesis of microbial flora is a complex process, and the optimization of medium composition and culture conditions is an indispensable part of the construction of microbial system. At present, most of the optimization of microbial culture mediums and culture conditions adopts single factor experiments, combined with orthogonal experiments or response surface experiments, to obtain optimized conditions efficiently. The medium is the basis of microbial reproduction and metabolism. The number of microorganisms and the production of antimicrobial active substances are affected by the composition of the medium and the culture conditions. Li et al. optimized the influencing factors of *Bacillus velezensis* ZJ20 strain by response surface and found that when the culture conditions were beef extract 3 g, peptone 10 g, sodium chloride 5 g, agar 20 g, distilled water 1 L, pH 7.0–7.2, it was most conducive to the growth of *Bacillus velezensis* [62]. Different types of strains have different preferences for the composition of the medium. The study of Shi et al. showed that *Bacillus velezensis* YH18 prefers the nitrogen source of the medium to the phytogenic source [63]. This conclusion is consistent with the results of Wang et al.’s optimization of the nitrogen source of *Bacillus megatherium* C_2 [64]. This may be because *B. velezensis* YH-18 is derived from cherry branches, so it is more sensitive to plant-derived nitrogen sources; it may also be because the nutrient composition of a plant-derived nitrogen source is higher than that of ordinary organic nitrogen sources and inorganic nitrogen sources, which is more suitable for the growth of strain YH-18 at a certain content. Touratier et al. showed that the ratio of C and N in the medium was also one of the important factors affecting the growth and metabolism of the bacteria [65]. Glucose, yeast extract and dipotassium hydrogen phosphate are commonly used carbon sources, nitrogen sources and inorganic salts in the cultivation of strains. They provide the necessary energy and nutrition for the growth of microorganisms. Kim et al. showed that the protease production of *B. subtilis* PANH-765 strain reached the highest level under the conditions of a medium with 2.0% glucose, 1.0% yeast extract, 0.2% ammonium nitrate, 0.2% ferrous sulfate and 0.2% dipotassium hydrogen phosphate [66]. In this experiment, the mixed strains tended to use glucose as the carbon source, yeast extract powder as the nitrogen source, and dipotassium hydrogen phosphate as the inorganic salt. The results of an orthogonal test showed that when the concentrations of glucose, yeast extract and dipotassium hydrogen phosphate were 3%, 2% and 1%, respectively, the growth effect of the mixed strain was the best, and the bacteriostatic rate of *FOC* 72-1 was the largest. The above results have an important reference role for the subsequent mixed strains in improving the field biological effects and environmental adaptability.

The strains can reproduce and produce spores rapidly under suitable culture conditions, but the optimum culture conditions between different strains are quite different. Therefore, the rapid propagation of specific strains can achieve the optimal growth conditions of the strains by optimizing the culture conditions. In this study, the cultivation of microbial communities requires higher rotation speeds and lower inoculation amounts, that is, the compound strain may have a strong oxygen consumption capacity. In order to create a good oxygen environment for the bacteria, the rotation speed and ventilation rate can be appropriately increased in the process of cultivating the microbial community [67], that is, increasing the dissolved oxygen to promote the growth and reproduction of the bacteria. It has been reported that the optimum fermentation conditions of *Bacillus velezensis* strain Xe01 were as follows: pH 7.0, temperature 30 °C, rotation speed 180 r/min [68]. Other studies have shown that the best combination of fermentation conditions for *Bacillus amyloliquefaciens* B1619 is as follows: temperature 28 °C, rotation speed 180 r/min, liquid volume 60 mL/250 mL. The efficiency is 35.4% higher than the initial culture conditions [69]. The results showed that the optimized culture conditions of the synthetic microbial community were similar to the rotation speed and temperature in many studies on the optimal culture conditions of *Bacillus*. By optimizing the culture conditions of *Bacillus amyloliquefaciens* HF-01, Hong et al. found that the fermentation broth with an inoculation amount of 2–12% had a certain antibacterial activity, and when the inoculation amount was more than 6%, the amount of viable bacteria began to decrease [70]. The amount of inoculation will affect the number of viable bacteria produced by the strain during the fermentation process and the control of related metabolites. If the inoculation amount is too large, that is, the initial number of bacteria is high, the nutrition is rapidly consumed, the cell value-added multiple is reduced, the metabolism is affected accordingly, and the number of viable bacteria decreases. Too little inoculation will prolong the fermentation period and reduce the productivity [71]. The optimum initial inoculation amount of the three strains was 4%, which was almost consistent with the conclusion of the optimum inoculation amount of *Bacillus* in most studies. The optimum inoculation amount of *Bacillus subtilis* J-4 was 5% [72]. The optimum inoculation amount of n-propanol *Bacillus cereus* isolated from liquor Daqu and fermented grains by Tang et al. was 4.9% after the optimization of fermentation process [73]. The optimum inoculation amount of *Bacillus mucilaginosus* was 5% [74]. The optimum inoculation amount of *Bacillus thuringiensis* JQD117 was 3% [75]. In this study, when the inoculation amount of mixed bacteria was 50–100 μL, the OD_600_ value increased with the increase in inoculation amount, which may be because the mixed bacteria grew faster at the beginning and the multiplication space was large. When the inoculation amount was between 100 μL and 200 μL, the growth rate of OD_600_ value decreased slightly, and the overall difference in inhibition rate was not significant. When the inoculation amount was 200–250 μL, the growth rate of OD_600_ value and the inhibition rate of *FOC* 72-1 were significantly reduced. This may be because with the increase in the number of bacteria, space and resources began to be limited, the competition between bacteria was enhanced, too much metabolic waste was produced, and the growth gradually began to be inhibited. Therefore, the inoculation amount of the mixed bacterial solution under the optimal growth conditions obtained by the response surface test was 200 uL, which was in line with the above theory. If the subsequent compound microbial agent needs to be used for fermentation production, the inoculation amount can be reasonably adjusted according to the actual situation. If the fermentation time is prolonged due to the small inoculation amount, the inoculation amount can be appropriately increased to shorten the fermentation time and save cost. In addition to the effects of rotation speed and inoculation amount, the effects of culture temperature, pH and culture time in the synthetic microbial community are also crucial. The results showed that the OD_600_ density of the synthetic microbial community increased first and then decreased with the increase in culture temperature. The possible reason was that when the culture temperature was lower than 30 °C, the mixed bacteria solution had an insufficient metabolic capacity due to the low temperature, which reduced the density of the mixed bacteria solution and affected the antibacterial rate of *FOC*. On the contrary, when the culture temperature is higher than 30 °C, the mixed bacterial solution may accelerate the decay of the bacteria due to the high temperature, and the OD_600_ density value also decreases, which makes its antibacterial effect on *FOC* poor. The pH value mainly affects the metabolism of microorganisms from two aspects: the charge state on the cell membrane of microorganisms and the enzyme activity. In this study, the optimum initial pH value of the mixed bacterial solution was 7. When the pH was lower or higher than 7, the OD_600_ value and antibacterial rate of the mixed bacterial solution were relatively low. The above results show that the mixed bacterial solution has a large suitable growth range for pH. However, when the growth environment of the mixed strain is acidic or alkaline, it is not conducive to its growth. The possible reason is that the pH value affects the permeability of the cell membrane, changes the ability of cells to absorb and excrete external substances, or affects the metabolic pathway of microorganisms by changing the activity of related enzymes, thus inhibiting the growth of strains. Therefore, after optimization, the neutral solution with an initial pH of 7 was more suitable for the growth of the complex microbial community. The optimal culture conditions determined by Pan et al. using a response surface optimization test was as follows: 52% liquid volume, culture temperature 28 °C, initial pH of 7, and 1% bacterial inoculation volume. After optimizing the culture conditions, the inhibition rate of LYMC-3 fermentation filtrate diluted by five times was 81.23%, which was 15.84% higher than that before optimization [76]. Sethi et al. optimized the culture conditions, such as pH, to achieve the maximum cellulase production of *Pseudomonas fluorescens*, *Bacillus subtilis*, *Escherichia coli* and *Serratia marcescens* [77]. Dong et al. studied the optimal fermentation conditions of *Bacillus licheniformis* SH003 using a single factor experiment: initial pH 6.5, growth temperature 35 °C, shaking speed 220 r/min, fermentation time 36 h [78]. The study by Wang et al. showed that the optimal culture conditions of *Bacillus megaterium* C_2 were temperature 37 °C, initial pH 7.0 and culture time 48 h [64]. In this study, the optimal culture time of the synthetic microbial community obtained by the response surface test was 21.6 h, which was significantly shorter than the fermentation time of other single strains. The results showed that there may be potential synergy between the three strains, which can promote each other’s growth. In summary, after the optimization of the conditions, the antibacterial activity of the composite bacterial solution is enhanced, and the method of optimizing the formula is feasible.

The response surface analysis method has the characteristics of shorter durations, a short cycle and high precision. It can overcome the disadvantages of a single factor test and is an effective method to optimize the fermentation conditions of the medium [79]. Tang et al. used response surface methodology to optimize the culture medium and culture conditions of *Lactobacillus* plantarum capsicum juice. The optimal culture conditions were determined as follows: pH 6.5, temperature 35 °C, inoculation amount 2%. Under these conditions, the bacterial concentration could reach 4.9×10^10^ CFU·mL^−1^ [80]. In this study, the significant influencing factors of mixed bacteria fermentation were analyzed by a response surface test; the inoculation amount, rotation speed and culture time, and the optimal fermentation parameters were obtained. The fermentation product will increase with the increase in inoculation amount and fermentation time. However, when it increases to a certain amount, it will inhibit the growth of the flora due to the reduction in resources and the accumulation of toxic substances. The rotation speed will have a certain impact on the metabolic activity of the strain. If the rotation speed is too fast, it may cause a certain pressure on the metabolic activity [81]. Therefore, the above three factors affect each other, and the best culture conditions need to be determined through experiments. All in all, in this experiment, the improved LB was used as the medium. On the basis of a single factor experiment, the inoculation amount, culture time, cultural temperature, culture speed and initial pH were selected as independent variables, and the OD_600_ and antibacterial activity of the compound bacteria solution were used as the response values. The response surface analysis was carried out. The optimal fermentation culture parameters were optimized according to the actual production: initial pH 7.0, cultural temperature 30 °C, inoculation amount 200 µL, and culture speed 171 r·min^−1^. Under these conditions, the OD_600_ value of the fermentation broth reached 3.16. In the later experiment, this model was verified, and the OD_600_ value obtained was 3.17, which was similar to the result obtained by the model prediction value, indicating that the model can be used in the later production practice. This result proves that the response surface method has an excellent effect in improving the optimization efficiency and reducing cost. The optimized synthetic microbial community in this paper can provide new biological resources for the research and development of biological agents in the later stage and is expected to be put into industrial production.

In summary, *Bacillus velezensis* VJH504, *Paenibacillus polymyxa* PJH16 and *Bacillus subtilis* JNF2 can produce metabolites that inhibit the growth of pathogenic bacteria and promote seedling growth. The microbial flora synthesized by the three strains has a stronger biocontrol ability than the single strain, which indicates that the synthetic microbial community may also have potential inhibitory effects on other pathogens, except for the pathogen *FOC* 72-1 in this study. This result has a certain guiding significance for the research and development of compound microbial agents for the efficient prevention and treatment of plant diseases. However, in the process of construction, the synergistic mechanism between microorganisms and whether they can inhibit other pathogens have not been explored. The next step will be to study the interaction mechanism between the three strains and whether the synthetic microbial community can control the *Fusarium* wilt of other crops, so as to develop a compound microbial agent for the efficient control of crop *Fusarium* wilt and promote its application.

## 5. Conclusions

In this study, the ratio of three strains of synthetic microbial flora for disease prevention and growth promotion was determined by an orthogonal experiment. The inoculation amounts of *B. velezensis* VJH504, *P. polymyxa* PJH16 and *B. subtilis* JNF2 strains were 4%, 4% and 4%, respectively. The fermentation basic culture of synthetic microbial flora was optimized by a single factor experiment and orthogonal experiment. The optimized fermentation basic medium was 30 g glucose, 20 g yeast extract powder, 10 g dipotassium hydrogen phosphate and 1000 mL double distilled water. The culture conditions of the synthetic microbial flora were optimized by a single factor experiment and response surface experiment. The final culture conditions were as follows: temperature of 30 °C, inoculation amount of 200 µL, culture speed of 171 rpm, culture time of 22 h, and initial pH of 7.0. Through verification, the results show that the above screening results are reliable. Pot experiments showed that the synthetic microbial flora had a better biocontrol effect than a single strain, which is of great significance for the development and application of compound microbial agents for the prevention and treatment of cucumber *Fusarium* wilt in the future.

## Figures and Tables

**Table 1:** Orthogonal test factor level table of initial inoculation concentration of strains.

Inoculation Level (%)	Factors		
	*B. velezensis* VJH504 (A)	*P. polymyxa* PJH16 (B)	*B. subtilis* JNF2 (C)
1	4	4	4
2	8	8	8
3	12	12	12

**Table 2:** Orthogonal test factor level table of five different concentrations of carbon source, nitrogen source and inorganic salt.

Concentration (%)	Factors		
	Carbon Source (D)	Nitrogen Source (E)	Inorganic Salt (F)
1	0.3	0.3	0.3
2	0.5	0.5	0.5
3	1.0	1.0	1.0
4	2.0	2.0	2.0
5	3.0	3.0	3.0

**Table 3:** L_9_ (3^4^) orthogonal experimental analysis table of the initial concentration of strain inoculation.

Experiment Number	Factors			Cell Density OD_600_	*FOC* Inhibition Rate
	A (*B. velezensis* VJH504)	B (*P. polymyxa* PJH16)	C (*B. subtilis* JNF2)		
1	4%	4%	4%	1.457	93.78%
2	4%	8%	8%	0.203	87.96%
3	4%	12%	12%	0.775	90.77%
4	8%	4%	8%	1.219	92.61%
5	8%	8%	12%	0.637	90.36%
6	8%	12%	4%	0.414	89.21%
7	12%	4%	12%	1.144	91.80%
8	12%	8%	4%	1.001	91.08%
9	12%	12%	8%	0.739	90.75%

**Table 4:** L_25_ (5^6^) orthogonal experimental analysis table of carbon source, nitrogen source, and inorganic salt ratios.

Experiment Number	Factors			Cell Density OD_600_	*FOC* Inhibition Rate
	D (Glucose)	E (Yeast Extract)	F (Dipotassium Phos-Phate)		
1	0.3%	0.3%	0.3%	1.279	80.68%
2	0.3%	0.5%	0.5%	1.495	81.79%
3	0.3%	1.0%	1.0%	1.355	80.99%
4	0.3%	2.0%	2.0%	1.528	82.64%
5	0.3%	3.0%	3.0%	1.477	81.41%
6	0.5%	0.3%	0.5%	1.253	80.12%
7	0.5%	0.5%	1.0%	1.312	80.77%
8	0.5%	1.0%	2.0%	1.592	84.11%
9	0.5%	2.0%	3.0%	1.560	83.56%
10	0.5%	3.0%	0.3%	1.923	88.06%
11	1.0%	0.3%	1.0%	1.180	79.68%
12	1.0%	0.5%	2.0%	1.819	86.52%
13	1.0%	1.0%	3.0%	1.943	88.42%
14	1.0%	2.0%	0.3%	2.041	92.79%
15	1.0%	3.0%	0.5%	2.001	89.89%
16	2.0%	0.3%	2.0%	1.633	85.33%
17	2.0%	0.5%	3.0%	0.596	75.19%
18	2.0%	1.0%	0.3%	2.058	93.01%
19	2.0%	2.0%	0.5%	2.057	93.01%
20	2.0%	3.0%	1.0%	1.674	90.13%
21	3.0%	0.3%	3.0%	2.003	89.89%
22	3.0%	0.5%	0.3%	2.125	93.47%
23	3.0%	1.0%	0.5%	1.096	78.21%
24	3.0%	2.0%	1.0%	2.222	94.89%
25	3.0%	3.0%	2.0%	0.827	77.69%

**Table 5:** Response surface three-factor three-level test design.

		Levels		
Encode	Factors	+1	0	−1
A	Inoculation amount (µL)	100	150	200
B	Culture speed (rpm)	150	175	200
C	Culture time (h)	18	21	24

**Table 6:** Response surface analysis scheme and test result.

Experiment Number	Factors			Cell Density OD_600_
	A (Inoculation Amount)	B (Culture Speed)	C (Culture Time)	
1	1	1	0	2.352
2	0	−1	1	2.685
3	1	0	−1	1.566
4	0	0	0	2.961
5	−1	0	1	1.598
6	−1	−1	0	1.949
7	0	0	0	3.287
8	0	0	0	2.873
9	0	0	0	3.351
10	0	−1	−1	0.475
11	−1	0	−1	1.011
12	1	0	1	1.935
13	0	0	0	3.497
14	1	−1	0	2.747
15	0	1	−1	0.726
16	0	1	1	1.154
17	−1	1	0	0.397

**Table 7:** Analysis of variance for regression model of the compound microbiome.

Source	SS	F Value	*p* Value	Significance Level
Model	15.98	11.06	0.0023	**
A—inoculation amount	1.66	10.34	0.0148	*
B—culture speed	1.30	8.10	0.0248	*
C—culture time	1.61	10.05	0.0157	*
AB	0.33	2.08	0.1922	
AC	0.012	0.074	0.7935	
BC	0.79	4.94	0.0616	
A^2^	1.19	7.43	0.0295	*
B^2^	2.69	16.77	0.0046	**
C^2^	5.41	33.69	0.0007	**
Residual error	1.12			
Lack of fit	0.84	3.98	0.1078	not significant
Pure error	0.28			
Total regression	17.11			
R-squared	0.9343			
Adj R-squared	0.8498			

* showed that the effect was significant (*p* < 0.05). ** showed that the effect was extremely significant (*p* < 0.01).

**Table 8:** Validation of experimental results.

Experimental Condition	Response Surface to Predict the Best Conditions	Verify the Best Conditions for Response Surface Prediction
Inoculation amount	200 µL	200 µL
Culture speed	171 rpm	171 rpm
Culture time	21.6 h	22 h
Cultural temperature	30 °C	30 °C
Initial pH	7.0	7.0
OD_600_	3.16	3.17

## Data Availability

The original contributions presented in the study are included in the article, and further inquiries can be directed to the first author (F.Y.).
